# The role of community and culture in abortion perceptions, decisions, and experiences among Asian Americans

**DOI:** 10.3389/fpubh.2022.982215

**Published:** 2023-01-17

**Authors:** Sruthi Chandrasekaran, Katherine Key, Abby Ow, Alyssa Lindsey, Jennifer Chin, Bria Goode, Quyen Dinh, Inhe Choi, Sung Yeon Choimorrow

**Affiliations:** ^1^Ibis Reproductive Health, Cambridge, MA, United States; ^2^National Asian Pacific American Women's Forum, Atlanta, GA, United States; ^3^Division of Complex Family Planning, Department of Obstetrics and Gynecology, University of Washington, Seattle, WA, United States; ^4^Ibis Reproductive Health, Oakland, CA, United States; ^5^Southeast Asia Resource Action Center, Washington, DC, United States; ^6^HANA Center, Chicago, IL, United States

**Keywords:** abortion, Asian American, AANHPI, sexual and reproductive health, immigrant health, medication abortion

## Abstract

**Introduction:**

Culture and community can play a role in views, stigma, and access related to abortion. No research to date has documented the influence of culture and community attitudes on Asian American (AA) experiences accessing abortion care in the United States (US). This paper aims to fill gaps in research and understand how cultural and community views influence medication abortion access and experiences among AAs.

**Methods:**

We used a community-based participatory research approach, which included collaboration among experts in public health, advocates, practitioners, and community partners to understand abortion knowledge, attitudes, and experiences among AAs. Using a semi-structured interview guide, we interviewed twenty-nine eligible people of reproductive age over 18 that self-identified as Asian American or mixed race including Asian American, Native Hawaiian, and/or Pacific Islander (AANHPI), and had a medication abortion in the US between January 2016 and March 2021. Interviews were analyzed and coded in NVivo 12 using a modified grounded theory approach.

**Results:**

Participants described various influences of religion negatively impacting acceptability of abortion among their family and community. Lack of openness around sexual and reproductive health (SRH) topics contributed to stigma and influenced most participants' decision not to disclose their abortion to family members, which resulted in participants feeling isolated throughout their abortion experience. When seeking abortion care, participants preferred to seek care with providers of color, especially if they were AANHPI due to past experiences involving stigma and judgment from White providers. Based on their experiences, respondents recommended ways to improve the abortion experience for AAs in the US including, (1) more culturally aware abortion providers from one's community who better understand their needs; (2) clinics providing abortion services located in or near AA communities with signage in local languages; and (3) tailored mental health resources with culturally aware therapists.

**Conclusion:**

This study demonstrates ways in which culture and community opinions toward SRH can influence both the acceptability of abortion and experiences seeking abortion care among AAs. It is important to consider family and community dynamics among AAs to better tailor services and meet the needs of AAs seeking abortion care in the US.

## Background

Asian Americans (AA) in the United States (US) are estimated to make up about 7% (around 24 million individuals) of the total US population (1). AAs are the fastest growing racial group in the US (2). Approximately two-thirds of the AA population in the US are immigrants and overall, AAs are projected to be the largest immigrant group in the US by 2050 (3). The AA community is extremely diverse (2), with members representing 50 distinct ethnic groups that speak more than 100 languages and dialects (4, 5).

Despite being a diverse racial group, research often fails to appropriately represent AAs by omitting AAs in health-related research or presenting aggregated AA data, which ignores differences in ethnic sub-groups. In cases where research is conducted among specific AA sub-groups, findings may be extrapolated and presented in a way that attributes findings to all AAs (6). This results in the health concerns of Asian American, Native Hawaiian, and Pacific Islanders (AANHPIs) being often underestimated or invisibilized in healthcare, policy, and advocacy spaces (7). Additionally, anti-Asian racism in the US (8), which has increased during the COVID-19 pandemic (9), also impacts health equity and access for AAs by presenting at three levels: individual (relating to how individual lived experience is impacted by racism), cultural (relating to how racist norms are embedded within our cultural beliefs and attitudes), and systemic (relating to societal structure that perpetuate racial inequality) (10).

Limited research exists related to AAs accessing sexual and reproductive health (SRH) care, and what research does exist, has focused on cervical cancer screening (11, 12) and the discriminatory nature of sex-selective abortion bans, which are based on the stereotype that AAs might prefer sons over daughters (evidence has shown that this is not true in the United States and AAs tend to have more daughters than sons than white Americans) (13, 14).

Looking at abortion incidence among AAs in the US, demographic data on US abortion patients collected by the Guttmacher Institute in 2008 found that 7% of respondents identified as AA and of the foreign-born respondents, 23% were Asian or South Asian (15, 16). A recent study on abortion rates among AAs in New York City, using data from the American Community Survey from 2011 to 2015, found that although the aggregate abortion rate per 1,000 women for people who identify as AA in New York City was 12.6, the abortion rate for each disaggregated group varied (17). In particular, the abortion rate for Indians (30.5) was much higher than the aggregate rate while the rate for Korean people (5.1) was much lower. Furthermore, Wu and Ada (2018) also found that AA subgroups differ significantly in their views toward legalized abortion (18). Groups ranked by the level of support for legal abortion (starting with most favorable and ending with least favorable) are: Japanese, Chinese, Asian Indians, Korean, Filipino, and Vietnamese Americans.

## Role of culture and community in abortion stigma

Culture and community can play a role in abortion stigma, which, in turn, can negatively impact people's experiences seeking and accessing abortion care and lead to decreased reproductive autonomy (19). Studies have shown that community stigma toward abortion arises as a result of cultural norms, including religious and gender norms. Religiosity, in particular, has been shown to be associated with higher abortion stigma (20–24). Additionally, gender norms that contribute to abortion stigma include stigma against women for engaging in sex outside of marriage as well as the view that motherhood is a defining characteristic of female gender identity (25, 26). Research has shown that other factors that intersect with gender, such as age and marriage status, similarly influence how someone who has an abortion is perceived—with stronger biases against young, unmarried people who seek abortions (25). It has been documented that for AA populations specifically, stigma related to sex and sexual and reproductive health (SRH) topics is common in families—which may feed into more negative abortion attitudes (27), however, stigma explicitly around abortion in AA communities has not been documented in previous literature. Additionally, beliefs about the acceptability of sexual education and SRH services, gender and religious norms, the role of parents and partners in the decision-making process, and taboos around premarital sex are all factors that can influence abortion care seeking experiences (28–31).

Abortion stigma, along with cultural context, can create secrecy around abortion thereby dissuading people from disclosing their abortion experience, which can in turn, prompt people to undergo this process by themselves to avoid disclosure (32–34). Going through the abortion process alone can further contribute to feelings of stigma for the individual seeking an abortion (35).

Within AA communities, we know that there exists a lack of openness among households in discussing SRH topics, especially with young people, which could have implications for care (36). Differing cultural contexts between parents and children, the specific influence of mothers (including their traditional cultural values) on their daughters' sexual behavior, and acculturation have been shown to influence sexual behavior—this can be especially prevalent among immigrant communities (36–47). Within AA communities, it is unclear if or how the abovementioned cultural factors relate to abortion. In this paper, we aim to address this gap by exploring the different ways in which community and culture inform views around abortion, influence stigma around abortion, and impact abortion experience among AAs.

## Methods

As a part of a larger mixed-methods study examining AANHPIs experiences with medication abortion (MA), we utilized a community-based participatory research approach (CBPR), which incorporated the expertise of public health researchers, advocates, practitioners, and community partners, to understand AAs' abortion knowledge, attitudes, and experiences. The research was overseen by a community advisory board (CAB) composed of seven members from community-based organizations and experts in AANHPI issues. CAB members were selected to represent sub-groups of interest including, LGBTQ+ AAs, Southeast Asians, Pacific Islanders, as well as an abortion provider who identifies as and works with AANHPIs to ensure these voices and perspectives were centered and uplifted in this work. Following guidance from previously conducted research (48–51), the CAB advised on each step of the research process including study design, instrument development, recruitment, and interpretation of results.

People of reproductive age over 18, who self-identified as Asian American or mixed race including AANHPI, could speak in English, and had a medication abortion in the United States between January 2016 and March 2021 were eligible to participate in the study. We specifically recruited participants who had a medication abortion, rather than a surgical abortion to address aims of the larger study, which sought to understand AANHPI's knowledge of and experiences with medication abortion. With support from the CAB, we recruited participants using online methods such as social media platforms, listservs, and mailing lists. We aimed to recruit up to forty respondents to interview a diverse set of respondents and reach thematic saturation. Interested participants completed an eligibility screening form using an online data collection platform (Qualtrics). Based on programmed screening logic, eligible participants provided an email address or phone number, which members of the study team used for outreach. Study team members invited eligible participants to participate in a phone screen through which additional background information was collected. At this time, we also confirmed participants' interest in participating in an in-depth interview after speaking with a member of the study team and receiving additional information and scheduled interviews.

Participants completed interviews *via* phone or on Zoom, depending on the preference of the participant. For interviews on Zoom, participants had the option to use video or not depending on their level of comfort. Before starting the recording and the interview, participants provided verbal informed consent to participate in the study and to be audio recorded. Interviewers with experience in SRH research and who were trained in qualitative research methods used a semi-structured interview guide with probes to ask participants about their personal identity, community attributes (interviewers probed specifically about friends and family), community and family views on sexual and reproductive health topics, including abortion, how these views influenced their decision to have an abortion and their overall abortion experience, and how abortion care can be more culturally aware and inclusive. Upon completion of the interview, participants received a $50 gift card *via* Rewards Genius in appreciation of their time.

Interviews were audio recorded and professionally transcribed. A study team member then removed identifying information from all transcripts and performed a quality assurance check on four interviews (14% of eligible sample) to ensure transcripts accurately matched audio recordings. We followed an iterative coding process on Nvivo 12, in which trained researchers of color, including researchers who identify as AA, from the team reviewed transcripts and together developed a preliminary codebook. The researchers then used this codebook to separately code two transcripts and further refined the codebook as needed. Once we achieved more than 95% agreement for each code, study team members separately coded the remaining transcripts. We applied a modified grounded theory approach (52) to analyze these data including (1) identifying themes and ideas emerging from the data, (2) organizing themes according to preliminary codes, (3) iteratively refining and expanding codes as necessary, and (4) describing relationships and patterns across codes and interviews. To describe ethnic identities in this paper, we reviewed open-ended responses interested participants wrote in on the screening form when asked to describe their country of origin or ethnic roots. In some cases, participants also described their ethnic identity when telling the interviewer more about themselves at the start of the interview. When describing ethnic identities in this study, we used the language participants used to describe themselves in interviews as was available. Once the research team, which consisted of people of color, including the principal investigator (PI) and co-PI who both identify as AA, identified themes, we presented preliminary findings to CAB members to collaboratively interpret our results and build out recommendations to reflect programmatic, clinical, and communication strategies to best engage with AA communities. Allendale Investigational Review Board granted ethical approval for this study.

## Results

From September 2021 to January 2022, we conducted thirty-two interviews with people who identify as AA or mixed race including AANHPI and had a medication abortion in the US between January 2016 and March 2021. Three interviews were excluded from analysis because the participants had used medication abortion pills for miscarriage management. We included twenty-nine interviews in the final analysis. Of the twenty-nine participants, twenty-six identified as Asian and three identified as mixed race including AANHPI. The average age of participants was 28 with ages ranging from 20 to 43. Participants represented three regions in Asia including, East Asia, South Asia, and Southeast Asia. One participant, who identified as mixed race, but did not provide additional information about their ethnic origins, was residing in American Samoa at the time of the interview. Participants' self-reported ethnicities included, Bangladeshi, Cambodian, Chinese, Filipina, Indian, Japanese, Korean, Laotian, Pakistani, Taiwanese, and Vietnamese. Close to forty percent of participants were born outside the United States. A majority of participants who were born outside the US had moved to the US over 20 years ago, while two participants had immigrated <10 years prior. Using the Pew Research center definition (53), we can describe the immigrant generation of twenty-three participants, including eleven first generation immigrants (whom we know were born outside the US based on a screening question); ten second generation immigrants (who shared that at least one of their parents had been born outside the US); and two participants who were considered third generation or more. All participants had their abortion before 8 weeks gestation. Table 1 shows additional background characteristics of participants. We discuss below the results related to how culture and community shaped views, acceptability, and experiences of abortion.

**Table 1:** Sample characteristics.

	**Total sample** ***n* (%)**
	29 (100%)
**Age** ^*^	
20–24	11 (37.9%)
25–29	7 (24.1%)
30–34	6 (20.7%)
35–39	4 (13.8%)
40–44	1 (3.4%)
**Education** ^*^	
Some high school or less	0
High school degree or GED	5 (17.2%)
Trade or technical school degree	1 (3.4%)
College degree	20 (68.9%)
Graduate or professional degree	3 (10.3%)
**Nativity** ^*^	
US	18 (62.1%)
Outside the US	11 (37.9%)
**US region for abortion** ^*^	
Northeast	6 (20.7%)
Midwest	6 (20.7%)
South	7 (24.1%)
West	9 (31.0%)
US territory (American Samoa)	1 (3.4%)
**Represented Asian regions**	
Central Asia	0 (0%)
East Asia	12 (41.4%)
South Asia	7 (24.1%)
Southeast Asia	7 (24.1%)
**Generation** ^†^	
1^st^ generation	11 (37.9%)
2^nd^ generation	10 (34.5%)
3^rd^ generation or more	2 (6.9%)
Missing	6 (20.7%)

* Responses from screening questions.

† Responses as available from screening question about nativity and interview transcripts.

### Cultural and community attributes

Almost all participants described attributes specific to their culture or community, both within and outside their AA-specific communities, and how these attributes contributed to and interacted with their personal identity. Participants often described an internal struggle to balance traditional family views, expectations, and strong religious influence with “western” ideals. In some cases, participants who identified as second generation described not feeling “Asian enough” due to differences between cultural practices in the US and community views. Participants also mentioned stereotypes they experienced from others and how these stereotypes interacted with familial and internal pressure to live up to expectations. One participant who reported identifying as Southeast Asian (age 24) explained that people often assumed she was “*soft spoken and innocent”*. Another participant, who identified as Korean American (age 35), explained how being stereotyped as the “model minority” added to pressure surrounding expectations by saying “*the whole stereotype of … Asians, you've been so successful, you've done so well in America, so you don't have any struggles”*.

“*…I need a man to guide me through life and to be my life partner because I can't sustain myself, when me, I'm very much the opposite, and I think in the current generation of … second generation south Asian American women, we're trying to break out of that mold. We're breaking out of that stereotype.”-* ethnic origins in Pakistan and Bangladesh, age 24.

“*…the typical Indian girl thing where like they think that you're perfect, and you wouldn't do anything bad”—*ethnic origins in India, age 29.

When describing community and personal attributes, respondents also mentioned how others perceived their race or ethnicity. Participants often described perceptions of their race or ethnicity in relation to their “proximity to whiteness”. One participant (ethnic origins in India, age 24) said, “*I think how others perceive me, I definitely have been told that I act White. I don't know what that means exactly, but I certainly have that feeling. I definitely think that people notice my race and notice the fact that I am neither Black nor White, but I do think that I look ethnically ambiguous enough where people are like what are you, and these are questions that I've been asked before.”* Another participant (ethnic origins in Vietnam, age 24) said, “*though I have proximity to Whiteness I'm still very much treated like an other”*.

### Influence of community views on acceptability of abortion

Participants discussed the influence of community views and values on attitudes toward and acceptability of abortion. A majority of participants described the importance of religion among their community and family. One respondent shared that it was important to discuss abortion and how it relates to religion because people tend to associate Filipino cultures with Catholism and may mistakenly assume that people who identify as Filipino don't believe in the right to choice, but that was not the case. In almost all instances where participants described the relationship between religion and views of abortion, they described religion as having a negative influence on acceptability. Two South Asian participants, both Muslims, explained that within Islam, abortion is “*not in violation to religious beliefs because Muslims don't believe in life at conception. We don't… like the soul doesn't enter the fetus until about like 122 or 120 days, so it's not until like four months… I feel like most people know around four months that they are pregnant. But if you have an abortion before four months it's not murder because there's no life, there's no soul. So Muslims don't usually have an issue with abortion.”—*ethnic origins in Pakistan, age 28.

Other participants explained that, while they were raised in families with more conservative views, having a more progressive external community and access to media and information on the internet influenced the progressive views they currently hold. One participant also shared that their progressive views stem from the fact that, in their opinion, protestors at clinics and other openly anti-choice individuals tend to be white, which indicated to them that as an AA person, they should be pro-choice.

“*I will say like there is definitely a sense of social identity too that like a lot of anti-choicers you don't typically see people of color and especially Asian people among anti-choice groups, right? So like when you see clinic protestors, I will say in my experience when I see clinic protestors they largely are White. I don't think I've ever seen a Black or Brown or Asian person at like the clinic in Philly, which is pretty shocking, because this is a very diverse place, and yet you never see that. So I do think that there was a sense of social identity there to be like well, I'm not White, and so to me I think connecting anti-choice views to White people was very helpful in me being like well, I'm not them, so (laughs) I guess I'm this.”—*ethnic origins in Japan, age 24.

Some participants also described younger generations as being more open and explained that it is more common to discuss SRH topics, especially abortion, with people their age compared to people in their parents' or grandparents' generations.

“*I do not openly talk about that, because coming from the generation that my parents are from and their parents—once I speak out about being supportive of that, then who knows … what doors I can open to things that will make me uncomfortable. I have accepted my grandparents and how my parents were raised, and I've accepted that they've come from a totally different country where we come from, America, where everything is so different…”—*ethnic origins in the Philippines, age 27.

In nearly all interviews in which participants described interaction and communication with family members, they explained that SRH topics and abortion were not openly discussed. Even participants who had previously said that their families or communities were not against abortion still said that this was not a topic they discussed openly, demonstrating stigma surrounding these topics, even in cases where it is considered more acceptable among families.

Many participants also explained that sex before marriage was unacceptable in their family and community and often equated the stigma of getting pregnant out of wedlock with the stigma of having an abortion.

“*you know, anything that deals with sex, to be honest, it's very, very like hush-hush, taboo. … a girl gets pregnant outside of marriage it always is—like the girl is always the one who takes the blame. She's the one who is shamed. And it's just a huge stigma. And so I know that that is, even today, how Korean culture views pregnancy or, you know, abortion and sex, and I think a lot of those kind of beliefs still—it's still around, even in Korean-American cultures”—*ethnic origins in South Korea, age 35.

Participants also described perceptions related to pregnancy and abortion that they had heard in their communities, such as all pregnancy should end in life, and a lack of understanding that it was okay to not have a child because one was not ready. Another participant further demonstrated how these views led to fear during the abortion experience due to stigma and stories she had heard growing up related to abortion.

“*I think I had a lot of fear going into the abortion process since I think growing up I kind of—It's very—it was a very stigmatized topic, and I think I kind of thought there would be some permanent like damage done to me, or like my ability to conceive in the future”—*ethnic origins in China and Japan, age 24.

Additionally, although a majority of participants considered themselves pro-choice or pro-abortion, some participants expressed feelings of shame and guilt when discussing their own abortion experience. These feelings of shame and guilt were not in relation to the decision to have an abortion, which many participants made clear throughout the interview; rather, participants related these feelings to becoming pregnant in the first place. Participants often felt internal stigma and worried that others would view them as irresponsible.

“*It's just like I feel like super embarrassing that I had an unintended pregnancy with someone that I didn't want to create like a family with. I thought it was just very shameful. And I just—it was just really embarrassing, and so I hated myself because I was like I already—I had one, right? And then it was like I got myself into the same situation again and it could have been prevented if I was being smart about it, but like—yeah. It was just really embarrassing for me, for more of like having an unintended pregnancy than actually having the abortion I think.”—*ethnic origins in India, age 24.

### Disclosing abortion decision and experience with family

Due to lack of openness around discussing SRH topics within families, a majority of participants chose not to tell their parents about their decision to have an abortion and about the abortion itself. In most cases, participants assumed their parents would be upset, but explained that they could not confidently speak to how their parents would react given a lack of previous conversations related to the topic. Other reasons participants chose not to tell their family about their abortion included: fear of judgement, stigma related to having sex outside of marriage and getting pregnant, as well as stigma surrounding abortion.

“*But the community views about having—like getting pregnant while not married I think is what drove- that had more impact, I think. I was more concerned about what my family would think, especially my grandparents in India. It would have been awful. I just feel like it would not have gone well if they ever found out about that. So I think more of that kind of perspective of like getting pregnant like that, I wasn't really thinking about views about abortion, I wasn't thinking about it like that.”—*ethnic origins in India, age 24.

Some respondents shared that this lack of discussion on SRH issues extended to them never having a regular gynecologist growing up—and many respondents shared that not having a gynecologist resulted in them feeling lost about how to seek care when they recognized their pregnancy.

A few respondents who did share their abortion experiences with members of their family experienced different reactions. Some respondents described being shamed when they shared their abortion decision—one respondent was forced to talk to religious, pro-life people, and people who regretted their abortion, while another described her abortion decision being used against her by a family member in an unrelated circumstance. Another respondent mentioned that when she shared her pregnancy news with her family, she did not feel supported—because it felt like they made the decision for her to have the abortion, rather than help her think through her options. So, although they did not pose a barrier, they were not supportive in the way she would have liked. Others described different members within the family having different reactions—some supportive and some not.

Some respondents chose to not use their insurance to pay for the abortion because they were covered by their parents' insurance and did not want them to find out about the abortion. Another had to lie to their family about needing money for a security deposit in order to borrow the money for the abortion, and this led them to feel very guilty about using their parents' money. One respondent, who lived with her family, booked a hotel for the night that she was taking the abortion pills, because she did not want to go through the abortion at home.

Some mentioned not having anyone to turn to for advice, and having to lie to their family on the days they were going through the abortion, all of which they found challenging. Overwhelmingly, participants mentioned feeling lonely while undergoing their abortion because they were unable to seek the support of their family.

“*I really wanted the feeling of like an older person or like, yeah, someone who could be like a family member to like reassure me or just be like, yeah, this is what it's like or like you're going to be okay. But I didn't feel like that was accessible.”-* ethnic origins in Japan, age 32.

Most respondents described keeping their abortion a secret as burdensome, heavy, and isolating. As one respondent described,

“*I think that having to do all this—went behind their backs and like not telling them about it, it was just a lot to handle, just because like I feel like I had to be so secretive with it, about what I was doing with my time, and like my parents were very worried about me during this time already. They were calling me every day, so yeah, that was kind of hard.”—*ethnic origins in Taiwan, age 22.

Another respondent shared how having the abortion could be isolating, even if it took place close to their home.

“*Everybody has everything to say if you're having the baby, but if you're not having the baby it's—it's not something you talk about, and it's usually something that's not positive. And so I mean, even reaching out to the Planned Parenthood, it kind of felt like I had to reach outside of my community into somebody else's community, even though it was … in my city, and so it's actually in my community… It's almost like …. people who fly out to Mexico, have an abortion and come back because it's somewhere away from home and somewhere that you get your stuff done and you come back, and nobody would know the difference.”—*ethnic origins in China, age 29.

On the other hand, one respondent shared that even though they were keeping their abortion a secret, they felt empowered by their decision to have the abortion, putting themselves first for a change.

“*I always have to think about my family, my husband first… this is like the only time that I feel like I've made a decision for myself. But at the same time thinking about my family, but in a way where it's like I'm doing this for me and not for them kind of thing.”*—ethnic origins in the Philippines, age 27.

### Healthcare system interaction

Interaction with the healthcare system was a key part of the process that shaped people's experiences and differentiated responses between subgroups.

One respondent highlighted the difference in approach to healthcare utilization among Japanese communities.

“*I think my upbringing, whether it's like my parents' like values or just kind of the way the healthcare system works in Japan, in contrast to how people conceive of—receive—seeking healthcare here is like if something is going on, go get it looked at right away, like the cost, like what else, what better thing could you spend your money on, so like any little thing that I have going on, I do seek care, and I think the mentality here is like un*t*il it's literally killing you like don't by any means, right?” -*ethnic origins in China (raised in Japan), age 31.

This respondent shared being shamed and stigmatized for the number of appointments they had, which ultimately led them to never go back to that particular healthcare system for care.

Some respondents shared feeling stigmatized by healthcare professionals when seeking an abortion, for getting pregnant, choosing to abort, and/or having had prior abortions. Others shared being stereotyped based on how they looked and/or how old they looked—for instance, assuming they were weak (because of their petite build), or that they must be smart, young (and hence shaming them for getting pregnant/having an abortion), or that they were stupid (if they did not speak English well).

“*I wish that when I first went to the clinic I wasn't so judged by that doctor and like basically shamed by him. That was—it was already at the time shameful enough that I was requesting for abortion pills, so the fact that he was just double shaming me by, you know, giving me dirty looks and then saying like we can't help you here was really disappointing and just made the process like even more stressful.”—*ethnic origins in South Korea, age 30.

Respondents shared feeling more comfortable in healthcare interactions where their provider was a person of color, especially if they were AANHPI. Some respondents shared that the discomfort from being seen by a White provider stemmed from discriminating experiences that they themselves or their community have had where their concerns had been dismissed. Participants described feeling more welcome, heard, and seen, when interacting with a provider from their own community. Having people of color in other roles, especially women of color, in the healthcare setting also made respondents feel more comfortable about seeking care.

“*It was such a great experience because like I had dealt with the non-Asian doctors trying to say like oh, well that doesn't sound—that sounds made up, or that's not right, things like that. And it was kind of—it was almost like comforting to finally not have that and not see that. And I think—I believe the people working the front desk were also all like people of color too, whether they were Asian or Hispanic or anything, it was just kind of nice to see.”—*ethnic origins in Japan, age 24.

Some respondents shared that there was a general mistrust of doctors in their community. One respondent, however, shared their belief that since Filipino Americans make up a large portion of the medical community, there is not as much mistrust of doctors among Filipino Americans compared to other AA subgroups.

“*I think mistrusting doctors is not as common among Filipino Americans because Filipino Americans make up a large portion of the medical community… So I don't see that same level of mistrust… I think it is also different between specifically being Filipino American versus being Asian American.”*—ethnic origins in the Philippines, age 27.

However, a few respondents shared being subjected to stereotyping even though they went to a Black, Indigenous, or person of color (BIPOC) provider. Although there is so much diversity among AAs, participants remarked that the stereotypes are unilaterally applied to all communities. One respondent described being stigmatized by a provider who, like this respondent, was also South Asian.

“*It would have been really nice to have someone from the same background as me, but I say that with caution because my current OB/GYN is also South Asian and it's also like a weird dynamic as well with that…I also feel like I've received judgment from her as well, because I said on my chart that I've had two pregnancies and I just feel like she judged me for that… at times, I felt like being talked down to, like kind of like saying, oh, you should already know this type of thing… I feel like as my doctor, you should be helping me learn to make healthy decisions instead of making me feel stupid that I wasn't aware or whatever.”—*ethnic origins in India, age 24.

A few respondents discussed the lack of representation of AAs in the reproductive health space and in abortion stories and how these led to stereotypes being propagated.

“*I think that they were feeding into the stereotypes of what they—what like the cultural stereotypes of perceptions about family and timing of having children, and then also abortion. And I think a lot of that in part has to do with the fact that AAPI people, there's not a lot out there or there's not a lot of representation out there of them and their experiences around abortion care… There's like a disconnect in terms of training and education about cultural competency in OB/GYN and specific to AAPI cultures.”—*ethnic origins in the Philippines, age 43.

As one respondent put it*, “the face of reproductive justice is kind of White still”* (ethnic origins in Japan, age 32). While reproductive justice, defined as “the human right to maintain personal bodily autonomy, have children, not have children, and parent the children we have in safe and sustainable communities” (54) was created and is led by Black women, this respondent relates the lack of Japanese-American or Asian-American role models in the abortion narrative, despite having AA friends involved in abortion activism, to the reproductive justice framework.

### Participants' reflections on improving medication abortion experiences for AA populations

Respondents reflected in various ways about how abortion experiences might be improved for AA populations. One theme that emerged was racial and ethnic concordance between providers and patients. Participants recommended being cared for by providers from one's community who understood their needs and were culturally aware. This included understanding that they themselves as providers may hold biases and stereotypes and should work consciously to unlearn those views. One participant shared that providers should be aware of how people's ability to access care was linked to colonialism and systemic racism. Other respondents shared that they just wanted to be seen as a person needing abortion care, and not judged by how they looked and how old they looked. A few participants described wanting their provider to connect with them at a personal level by talking to them about their background and context.

Participants also reflected on the location of clinics offering abortion services—suggesting that more clinics be located in or near communities with Asian groups, with signage in local languages, and accessible to people with disabilities. One respondent shared that seeing signage in their local language made them feel safer and could help make abortion more acceptable in their community. Respondents also shared that having paperwork in their local language and/or translators and interpreters available at the clinic would signal more inclusive care, and be especially useful to immigrant communities. Another respondent shared that a “community hub”—where doctors and clinic staff were all from the same community as the people they were serving, and providing not only medical care but also serving as a place for the community to come together to celebrate cultural events and host gatherings—could also help local communities feel at home.

One respondent also suggested that Asian American advocacy organizations like the National Asian Pacific American Women's Forum (NAPAWF) could work with pro-choice providers or associations like Medical Students for Choice to sensitize them to the myriad cultures within AANHPIs and their differing perspectives on SRH and abortion, as a way to build a cohort of more culturally-aware providers.

Another participant shared that there was a need for more inclusive services for those who practice Islam.

“*I've never really experienced like a truly inclusive experience (laughs) being Muslim. And so I don't know what that would look like.”*—ethnic origins in India, age 32.

Although respondents shared that growing up with a narrative that demonized, sensationalized, and/or trivialized abortion resulted in them not seeking the mental health support they needed and taking longer to come to terms with their abortion, many respondents also highlighted the need for tailored mental health resources. Respondents described needing therapists who were culturally aware, had similar upbringing, could speak the same language, and have experienced an abortion. Support groups for people in the same community, either held in-person or virtually, was another recommendation from participants. One respondent also suggested that clients should be given the option to be a part of these groups and/or receive culturally-relevant reading material that would help them relate with others from their community who have also undergone the process and help “make sense of their experience”.

“*I've just had therapists who are White before who have said things like, oh, like just be very transparent and set boundaries with your parents, which is not really something that exists in Asian cultures …—I think some people at least like can feel very, very drained after an experience like this. I mean, help or therapy that is really irrelevant or not understanding just can make things worse really.” –* ethnic origins in South Korea, age 24.

Part of having culturally-inclusive mental health resources included understanding whether and when one may want to share their abortion experience with their family. One respondent shared that the clinic they went to forced mental health services on them—and the help offered lacked understanding around family dynamics and cultural expectations in their community, which led to questions and recommendations around talking to their family—something the respondent knew would have only been counterproductive. These experiences reflected the need for training and education for providers on aspects of Asian culture and how they may relate to care seeking.

## Discussion

Our findings begin to provide insight into the preferences, needs, and experiences of abortion care among AAs. Our findings indicate that AAs balance a tenuous relationship between wanting family support and acceptance but also establishing ways in which they are different from their parents and community. Maintaining good parental relationships and avoiding parental disappointment is a factor that has been shown to influence individual abortion access and experience in other contexts (55). We see this echoed in our findings among AAs as well. Despite not having open conversations about sex within families, our study indicates that AAs craved support from family members, demonstrating the importance that family holds among AAs. Additionally, respondents expressed wanting support from the time of abortion decision-making until well after the abortion procedure is completed. Given that AAs are not receiving the support they desire from families, counselors and mental health support professionals should work toward offering care throughout the abortion journey of their AA clients.

Additionally, AAs are more likely to live in multigenerational households with higher caregiving obligations, which may contribute to the fear of family judgement (56, 57). Living in closer proximity to and regularly providing care for family members makes it more difficult for someone to maintain their privacy when seeking an abortion, as was seen in the number of ways respondents navigated interactions with their family when preparing and undergoing their abortion. Hence, when offering mental health support, counselors should keep in mind the living situation of and socio-cultural expectations that come with being AA.

Most respondents described feeling isolated because they were unable to share their abortion with their parents/family. Particularly, many participants were unaware of how their families may react to the disclosure given that these topics were never discussed. Additionally, respondents seemed more concerned about the stigma around having sex than having the abortion itself. Our study indicates that abortion stigma may begin with the act of having sex, particularly outside marriage (and community/family members finding out about it), rather than with abortion decision-making, as most literature in the United States may indicate. One study in Indonesia refers to the “social value of virginity” and abortion dispelling the virginity status; (25) and another study explores the need among South Asian Americans to maintain their virtuous image within their community (20). Further exploration of how expectations around virginity interact with abortion stigma within the wider AA community is warranted.

Our study demonstrates that different groups within AAs view abortion through different religious and cultural approaches. As has been found in other studies (21, 26, 32), most of our respondents spoke about the influence that religion had on their abortion experience. South Asians tended to discuss community/culture influencing abortion stigma rather than religion, as compared to other groups. Muslims in our study, however, specifically discussed the role of Islam in impacting their view of abortion. These differences in perception highlight the diversity of beliefs and factors influencing abortion care within AAs.

Our study also adds to the growing literature on the impact of racism on health. As reported in other studies focused on communities of color (58), our respondents also sought out physicians who were AA or at least identified as a person of color with the hope that their experience would be better. Policymakers should implement strategies to build a diverse healthcare workforce that includes underrepresented minorities (58).

Similar to experiences of racism within the healthcare system that have been documented among Black (58) and Latinx (35) populations, the AAs in our study also experienced individual racism in their interactions with the healthcare system. Respondents recount their concerns being dismissed by White providers and their preference for providers of color, especially those from their own communities. It is striking to see the stereotypes that BIPOC providers, even those who identify as AA, propagate within their own communities, reflecting the lack of understanding among AAs about the diversity among their own communities. This highlights the need for more research, tools, and resources that center the diversity of AANHPI communities and showcases the differences in perspectives on, access to, and healthcare utilization of SRH services among subgroups, as well as cultural sensitization trainings for all providers.

Our findings demonstrate that stereotyping is not restricted to only the healthcare settings—respondents described being stereotyped in other aspects of life by those around them and being assessed by their proximity to being White. Additionally, our study findings indicate that like Black (58) and Latinx populations (35), AAs also experience gendered racism in expectations related to women being “soft spoken” and “innocent”. However, while Black and Latinx communities report being viewed by “negative” stereotypes (35), respondents in our study shared that there was an expectation for them to conform to the positively-viewed “model minority” myth. This highlights the need for efforts to break the stereotypes by lifting up the voices of AANHPI communities and have more representation of diverse AANHPIs in all walks of life, including media.

When discussing AA experiences, it is also important to highlight ways in which findings may be similar or different across ethnic groups. Research conducted with and among AAs and their health outcomes often aggregates various AA subgroups together, masking important differences between Asian ethnicities (6). Aggregation also ignores the varied social histories of the different subgroups, which impacts adversely how health outcomes among specific AA communities are understood (59). Such perceptions view AAs as a monolith, without acknowledging the disparities among subgroups. While we identified common threads in the way participants in this study described cultural and community influences on their abortion experience, some participants also discussed culture specific attitudes or practices that stood out from other AA experiences in this study. Other studies documenting AA health outcomes (60–63) and access to care (64, 65) with disaggregated data report have also reported differences between subgroups, including differences related to sexual and reproductive health (SRH) outcomes (8, 66). Understanding abortion experiences among different AA subgroups can help guide policy and practice related to abortion care that will better meet the needs of AAs in the US overall as well as the needs of AA subgroups.

Our study is not without limitations. We were unable to recruit respondents identifying as Native Hawaiian (NH) or Pacific Islander (PI), and only very few (*n* = 3) of our respondents identified as mixed race that may include NH or PI. Additionally, although close to forty percent of our respondents were born outside the United States, detailed differences in views and experiences were not captured in our study. Future research should include purposive recruitment of different subgroups to tease out the differences between AANHPI communities; and AANHPI immigrants (recent as well as across different generations) to understand the impact of acculturation on MA perspectives and access. Additionally, exploration of culturally appropriate, inclusive abortion care among Muslims in the United States, is also another area that needs further research. Lastly, our study has documented aspects of individual racism that respondents have experienced. Future work should aim to investigate how structural and cultural racism impact AAs. Finally, given the aims of the larger study, we only interviewed people who had a medication abortion so we cannot generalize these findings related to abortion experience to other types of abortions. Future work should seek to understand how culture and community influences expand to AA experiences with procedural or later abortions.

## Conclusion

Our study explored how community and culture shape and influence perspectives around and experiences with abortion in the AA community. Our findings contribute to a better understanding of AA communities' needs and preferences when accessing abortion. With the recent Supreme Court decision overturning Roe v. Wade (67), people of color, including AANHPIs are more likely to be adversely impacted. Our study shows that AAs already experience discrimination when seeking care. Policymakers, clinicians, mental health professionals, and advocates should work toward ensuring that AANHPIs receive the care they deserve, integrating aspects of community and culturally-inclusive care in their practice.

## Data availability statement

The raw data supporting the conclusions of this article will be made available by the authors, without undue reservation, upon request to the corresponding author.

## Ethics statement

The studies involving human participants were reviewed and approved by Allendale Institutional Review Board, United States. Written informed consent for participants was not required for this study to maintain participant confidentiality due to the sensitive nature of the study.

## Author contributions

SrC and SuC conceived and designed the study and secured funding for the work. SrC, KK, and BG were involved in data collection. SrC, KK, BG, AO, and AL contributed to data analysis. SrC, KK, and AO authored the manuscript. AL, JC, BG, and QD reviewed the manuscript and provided feedback. All authors contributed to the article and approved the submitted version.
